# Mis- or Missed Diagnosis: A Series of Four Cases

**DOI:** 10.1155/2012/946327

**Published:** 2012-07-02

**Authors:** Sheela Sreedharan, Bhat Sangeetha Govinda, Iyer Satishkumar Krishnan, Kumar Kavita Krishna

**Affiliations:** ^1^Department of Pedodontics and Preventive Dentistry, Government Dental College, 695011 Thiruvananthapuram, India; ^2^Department of Pedodontics and Preventive Dentistry, St. Gregorious College of Dental Sciences, Kottayam, India

## Abstract

Diagnosis forms the backbone of treatment planning. Accurate diagnosis is essential to initiate the appropriate treatment at the apt time. Diagnosis involves eliciting the signs and symptoms of the patient and their accurate interpretations. The subtle signs that can go unnoticed lead to misdiagnosis and subsequent agony to the patient. Alertness on part of the clinician is important to avoid this error. Reported in this paper are four cases that were wrongly diagnosed either due to lack of clinical experience or due to omission of careful clinical, radiographic, and histopathological examinations.

## 1. Introduction

A misdiagnosis is, simply put, a wrong diagnosis. An erroneous diagnosis can take a number of forms, from a missed diagnosis in which no medical problem is identified when a problem exists, to a diagnosis which later turns out to be wrong, such as classifying a benign tumour as malignant. Misdiagnosis is a form of medical error, and while it is difficult to get accurate statistics on wrong diagnoses, some statistics place the rate at around one to two percent, with varying consequences.

There are a number of reasons for a misdiagnosis to occur. People who have suffered as a result of a misdiagnosis are often tempted to blame lazy doctors or medical personnel, but all kinds of things can be involved, including malfunctioning medical equipment, a patient's decision to conceal information, a language barrier between doctor and patient, inexperience on the part of the doctor, or a situation in which a diagnosis is extremely unusual, making it hard for a doctor to recognize the signs. Sometimes a disease may also manifest in an unusual way, with a doctor excluding a diagnosis because the symptoms do not fit and later realizing that the patient's case was atypical.

## 2. Case Reports

Four cases which had been referred to the Department of Pedodontics and Preventive Dentistry, Government Dental College, Thiruvananthapuram, Kerala, India, have been described.

**Case 1**

**Figure 1:**
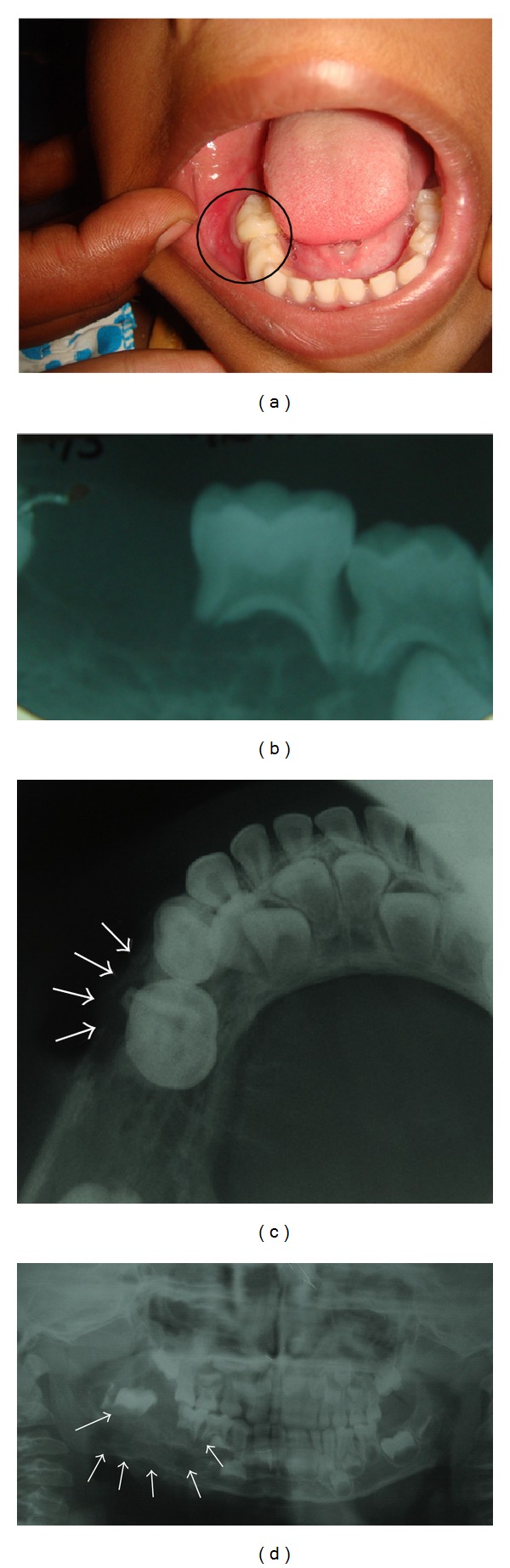
(a) Erythema of the vestibular mucosa in relation to 84 and 85. (b) Intraoral periapical radiograph showing resorption of roots of 84, 85, periapical radiolucency, and displaced tooth buds. (c) Mandibular occlusal radiograph showing erosion of the buccal cortex in relation to 85. (d) Panoramic radiograph showing lytic lesion over the right mandible. Note the irregular lower border of the mandible along with the displaced tooth bud of 46 (arrows).

A 5-year-old girl was referred from a general dental practitioner with a diagnosis of a periapical abscess with swelling, pain, and erythema of the gingiva in relation to teeth numbers 84 and 85 (Figure 1(a)) with an associated grade III mobility of 85. The IOPA (intraoral periapical) radiograph (Figure 1(b)) showed root resorption of 85 and displacement of the developing tooth bud of 46, due to a mixed radiolucent radiopaque lesion with ill-defined borders. The occiusal radiograph (Figure 1(c)) showed erosion of the buccal cortex. A destructive bony lesion of the mandible on the right side with displacement of the tooth buds of 46 and 47 was evident on the panoramic radiograph (Figure 1(d)). CT scan showed an expansile lytic lesion of the mandible with discontinuity of the buccal and lingual cortices. The developing first molar was found to be contained in the lesion. A hypothesis of malignant tumour was postulated. The child was referred to the Regional Cancer Centre in Thiruvananthapuram where the lesion was diagnosed as round cell tumour and treatment for the same commenced.

**Case 2**

**Figure 2:**
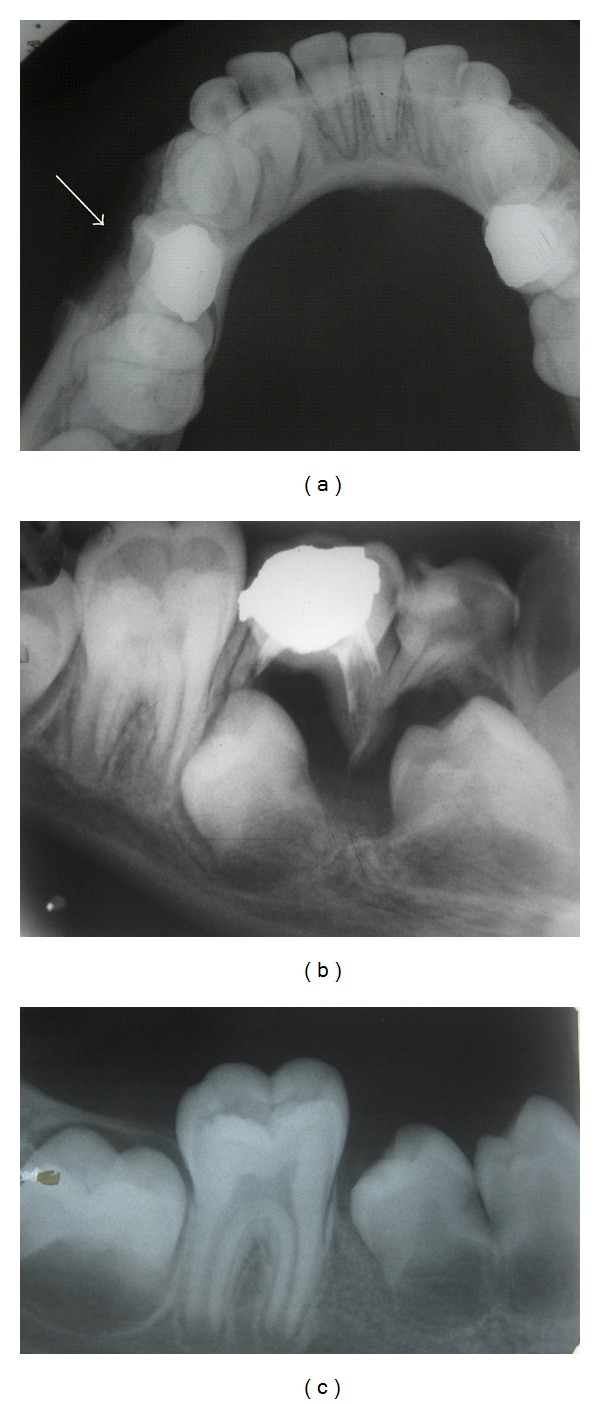
(a) Mandibular occlusal radiograph in case 2 showing erosion of the buccal cortex of the mandible. (b) Intraoral periapical radiograph showing radiolucency in relation to 84, 85 along with displacement of the underlying successor teeth. (c) Intraoral periapical radiograph showing eruption of 44, 45 following extraction of 84, 85 and periapical curettage.

A 5-year-old girl was referred with a diagnosis of Ewing's sarcoma. The child presented with a swelling of the right of the face of 2-week duration. On examination a fluctuant swelling was found in the buccal vestibule opposite 84 and 85. Pulpectomy of 85 had been done 2 1/2 years back. IOPA radiograph (Figure 2(b)) revealed a radiolucent lesion in the periapical region of 84 and 85 extending downwards till the developing premolar tooth buds leading to their displacement. An occusal radiograph (Figure 2(a)) showed erosion of the mandibular buccal cortical plate on the right side. Fine-Needle Aspiration Cytology (FNAC) of the lesion was done, which suggested it to be a suppurative lesion. Extraction of the involved teeth was done followed by through curettage. The histopathology report confirmed the lesion to be an inflamed odontogenic cyst.

**Case 3**

**Figure 3:**
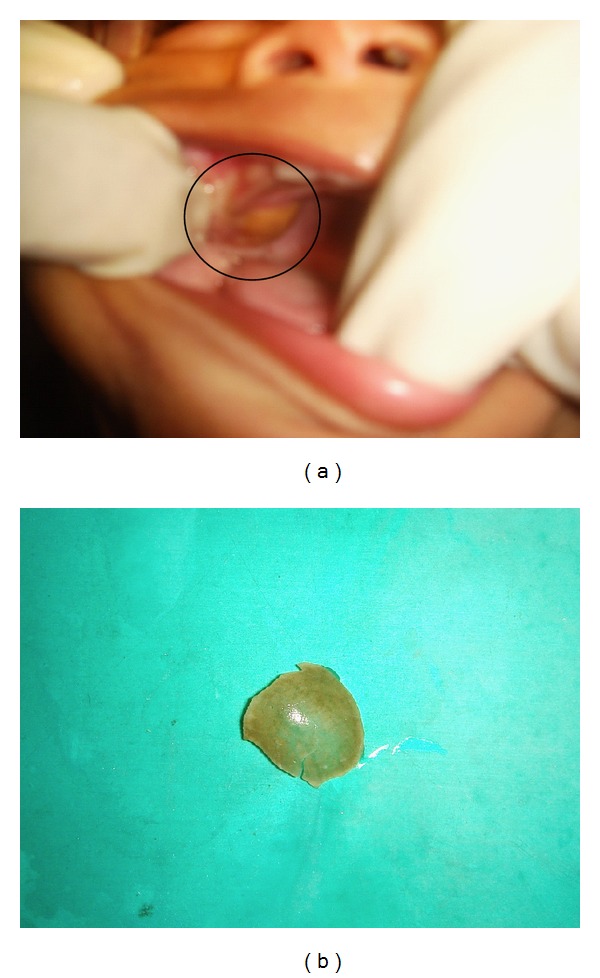
(a) Suspected neuroectodermal tumour of infancy. (b) Peel of a jackfruit seed that caused the illusion.

A 5-month-old infant was referred for biopsy with a diagnosis of neuroectodermal tumour of infancy. The parent had noticed a swelling on the palate 2 days before reporting to our department. A pigmented lesion was found on the palate on the right side (Figure 3(a)). On careful examination the patch was found to move during palpation. In the course of examination the lesion was excised and was found to be the peel of the seed of a jackfruit (Figure 3(b))!

**Case 4**

**Figure 4:**
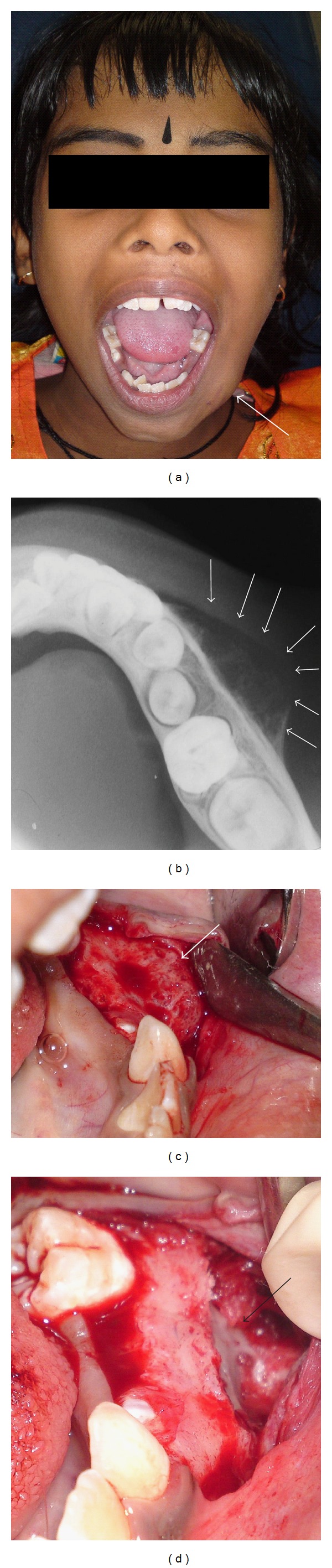
(a) Swelling of the mandible of 2-month duration. (b) Mandibular occlusal radiograph showing intact cortical plate and periosteal bone formation (arrows). (c) On surgical exposure, bony lesion seen. (d) Bone removed for biopsy.

An 8-year-old girl was referred with a diagnosis of ostetosarcoma of the left side of the mandible. The child presented with a bony hard swelling of the left side of the mandible in relation to teeth 74 and 75 (Figures 4(a) and 4(b)) of 2-month duration. There was no associated pain. On surgical examination the swelling was found to be bony, and the cortical plate of bone seemed to be intact (Figures 4(c) and 4(d)). Histopathological examination of the surgical specimen revealed it was sequestrum and the lesion was osteomyelitis!

## 3. Discussion

Dentists play an important role in the early diagnosis and treatment of oral lesions. Ideally, this involves not only a thorough oral and sometimes a radiographic examination to identify the condition, but also confirmation of the clinical impression by biopsy and subsequent microscopic evaluation. However, treatment based solely on a clinical impression of the diagnosis, without histologic confirmation, can result in serious consequences, particularly when the lesion is precancerous or cancerous.

In a study by Kondori, Mottin, and Laskin, of the clinical diagnoses made by the dentists submitting specimen for histopathological examination, 43% were incorrect. General dentists misdiagnosed 45.9%, oral and maxillofacial surgeons 42.8%, endodontists 42.2%, and periodontists 41.2% of the time. The most commonly missed clinical diagnoses were hyperkeratosis (16%), focal inflammatory fibrous hyperplasia (10%), fibroma (8%), periapical granuloma (7%), and radicular cyst (6%). Cancerous lesions were misdiagnosed 5.6% of the time [1].

A mistaken diagnosis or a wrong diagnosis can cause undue agony to the patient and the parent (in case of children) till establishment of the correct diagnosis. In the cases reported attention to detail could have prevented parental distress as well as delay in the treatment procedure.

Round cell tumours are rare destructive lesions of bone [2]. Children are the most frequently affected. Pain, rapid swelling, paraesthesia, and neuralgia may be early clinical signs of the disease. Very few cases have been reported in children below 5 yrs of age. Its occurrence in the head and neck region is unusual and when it occurs in the jaw, mandible is more frequently affected than the maxilla [3]. The tumour has an aggressive behaviour characterized by rapid growth and high probability of micrometastasis at diagnosis [4]. Brazăo-Silva et al. described a case of round cell tumour of the mandible which had been treated as a dental abscess and ended fatally [5]. In the first case described in this paper the child was sent to our department for management of the seemingly abscessed teeth. Here the radiographs clearly indicated that the lesion was not so innocent. The displacement of the tooth buds (Figures 1(b) and 1(d)) and erosion of the buccal cortex (Figure 1(c)) should have created suspicion in the mind of an alert clinician. The panoramic radiograph (Figure 1(d)) explains the destructive nature of the lesion. Further detailed examinations like the CT scan or an FNAC could have helped avoid the delay in treatment of the malignant condition.

The second case presented in this paper was diagnosed as Ewing' sarcoma and referred for biopsy. The clinical and radiographic picture was the same as that in case one except that the lesion was fluctuant. The general practitioner had seemed to miss this finding and had concluded that it was an Ewing's sarcoma. The fluctuant nature of the lesion led to an FNAC which suggested that the lesion was suppurative.

Melanotic neuroectodermal tumour of infancy (MNTI) is a relatively uncommon osteolytic-pigmented neoplasm that primarily affects the jaws of newborn infants. There is increased excretion of vanillylmandelic acid in children affected with the tumour due to the neural crestl origin [5]. In the third case the clinical picture with the markedly pigmented surface led the clinician to suspect neuroectodermal tumour of infancy. In our country it is common for the general dental practitioner to refer most of the child patients to the pedodontist. In the third case, more so as the child was young and uncooperative. This might be one reason why the general practitioner had not conducted further detailed examinations including palpation of the lesion resulting in the erroneous conclusion.

The fourth case was suspected to be an osteosarcoma, again the result of a hasty conclusion. The child had presented with a swelling of the left side of the mandible of 2-month duration. There was no associated pain. Osteosarcoma is a malignant bone tumour that usually develops during the period of rapid growth that occurs in adolescence, as a teenager matures into an adult. The associated signs and symptoms include bone pain, pathological fractures, and pain during function. Plain radiographs of conventional, intramedullary osteosarcoma most commonly show a mixed lytic and sclerotic lesion causing cortical destruction, often with an associated soft tissue mass that may contain calcification [7]. Various periosteal reactions may be present but the most common is the sunburst, spiculated type. This feature is nearly pathognomonic of osteosarcoma but can also be seen with osteoblastic metastasis. A Codman's triangle is common in osteosarcoma, but is less specific [7]. It is a term used to describe the triangular area of new subperiosteal bone that is created when a lesion, often a tumour, raises the periosteum away from the bone. The fourth case described in this series was an osteomyelitis suspected to be osteosarcoma. Osteomyelitis is the infection of bone and can be acute, subacute, and chronic. Radiographically evident bone destruction and periosteal reaction take 2-3 weeks to develop. Eventually metaphyseal lucencies with varying degrees of cortical destruction and periosteal new bone develop. The periosteal reaction can appear as lamellated, onionskin, or spiculated new bone or a Codman's triangle due to subperiosteal abscess. The mandibular occlusal radiograph, in the fourth case, showed subperiosteal bone formation and a more diffuse, mineralized osteoid forming within the soft tissues adjacent to the bone, a typical Codman triangle (Figure 4(b)), which led the general practitioner to believe that it was an osteosarcoma. But biopsy after a surgical exposure showed it to be an osteomyelitis.

 The parents of three of the children had been informed that the lesion was something with a malignant potential though the diagnosis had not been established nor had detailed examinations or lab tests been conducted. The parents were very apprehensive and agonised till the establishment of the final diagnosis. This is where reaching a definite diagnosis matters. As health care professionals it is utmost important that dentists give importance not just to the presence or absence of disease but also to the social and mental well-being of the patient and the parent.

## 4. Conclusion

 As a clinician one has to be careful in eliciting the patient's signs and symptoms. An accurate interpretation of these is even more important. Theoretical knowledge and clinical experience too matter in preventing a wrong diagnosis. But most importantly one has to weigh all the possible conditions that can manifest with the patients symptoms and signs before confiding with the patient, so that unnecessary emotional and mental trauma to the patient can be avoided.

## References

[1] Kondori I, Mottin RW, Laskin DM (2011). Accuracy of dentists in the clinical diagnosis of oral lesions. *Quintessence International*.

[2] VikaS PB, Ahmed MBR, Bastian TS, David TP (2008). Ewing’s sarcoma of the maxilla. *Indian Journal of Dental Research*.

[3] Lopes SL, Almeida SM, Costa AL, Zanardi VA, Cendes F (2007). Imaging findings of Ewing’s sarcoma in the mandible. *Journal of Oral Science*.

[4] Heare T, Hensley MA, Dell’Orfano S (2009). Bone tumors: osteosarcoma and Ewing’s sarcoma. *Current Opinion in Pediatrics*.

[5] Brazão-Silva MT, Fernandes AV, de Faria PR, Cardoso SV, Loyola AM (2010). Ewing’s sarcoma of the mandible in a young child. *Brazilian Dental Journal*.

[6] Borello ED, Gorlin RJ (1966). Melanotic neuroectodermal tumor of infancy—a neoplasm of neural crese origin. report of a case associated with high urinary excretion of vanilmandelic acid. *Cancer*.

[7] McCarville MB (2009). The child with bone pain: malignancies and mimickers. *Cancer Imaging*.

